# Using ^2^H labelling to improve the NMR detectability of pyridine and its derivatives by SABRE

**DOI:** 10.1002/mrc.4703

**Published:** 2018-01-25

**Authors:** Philip Norcott, Michael J. Burns, Peter J. Rayner, Ryan E. Mewis, Simon B. Duckett

**Affiliations:** ^1^ Department of Chemistry University of York York YO10 5DD UK; ^2^ Division of Chemistry and Environmental Science Manchester Metropolitan University Manchester Greater Manchester UK

**Keywords:** hyperpolarisation, NMR, parahydrogen, SABRE

## Abstract

By introducing a range of ^2^H labels into pyridine and the para‐substituted agents, methyl isonicotinate and isonicotinamide, we significantly improve their NMR detectability in conjunction with the signal amplification by reversible exchange process. We describe how the rates of T_1_ relaxation for the remaining ^1^H nuclei are increased and show how this leads to a concomitant increase in the level of ^1^H and ^13^C hyperpolarization that can ultimately be detected.

## 1 INTRODUCTION

**Scheme 1:**
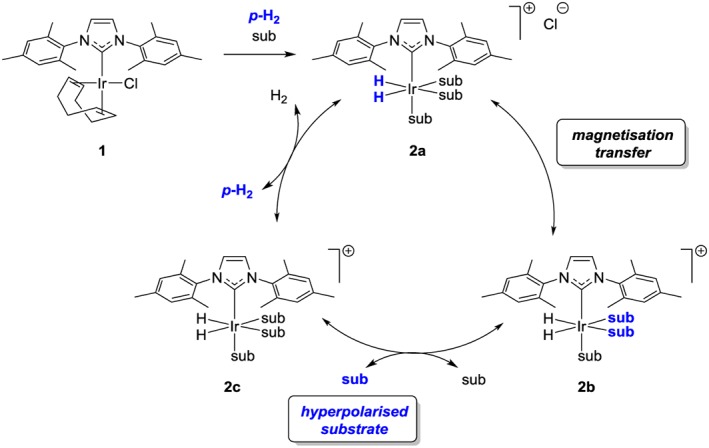
Schematic representation of the signal amplification by reversible exchange (SABRE) process wherein the substrate (sub) achieves hyperpolarization through interactions within the metal complex **2a**

Hyperpolarization techniques overcome the inherent insensitivity of nuclear magnetic resonance (NMR) and magnetic resonance imaging by manipulating the spin distribution across Zeeman‐split energy levels away from equilibrium where a typical population difference used to produce a magnetic resonance (MR) signal can be less than 1 in 100,000. The employment of hyperpolarized states can therefore result in very substantial MR signal gains. This process can be achieved through a number of methods, the most common of which are dynamic nuclear polarization^1^, ^2^, ^3^ and *para*hydrogen induced polarization.^4^, ^5^, ^6^ Signal amplification by reversible exchange (SABRE)^7^ has recently emerged as a powerful alternative that can rapidly and repeatedly hyperpolarize a substrate through the interaction of *para*hydrogen with an iridium catalyst. In SABRE, *para*hydrogen (*p*‐H_2_) adds to a precatalyst, such as [IrCl(COD)(IMes)] (**1**, where IMes = 1,3‐bis(2,4,6‐trimethylphenyl)imidazol‐2‐ylidene and COD = *cis*,*cis*‐cycloocta‐1,5‐diene), in the presence of a coordinating substrate (sub) to form an iridium hydride complex such as [Ir(H)_2_(IMes)(sub)_3_]Cl (**2**)^8^, ^9^ (see Scheme 1). Polarization from the *para*hydrogen derived hydride ligands is then transferred into the bound substrate through the *J*‐coupling network that exists in this complex.^10^ Throughout the process, *para*hydrogen and the substrate, which are located in the bulk solution, are in reversible exchange with the corresponding ligands that are bound to the iridium complex. This results in a build‐up of hyperpolarized substrate in bulk solution and hence the signal gain grows with *para*hydrogen exposure time, although a limit is reached because relaxation acts to destroy the hyperpolarization that has been created through SABRE.^11^ As the rates of ligand exchange are relatively fast, substantial hyperpolarization levels can be produced in just a few seconds. SABRE has been demonstrated for a wide range of substrates which include pyridine and its derivatives,^9^, ^12^, ^13^, ^14^, ^15^, ^16^ pyrazines,^16^, ^17^ imidazoles,^18^, ^19^ pyrazoles,^20^, ^21^ pyridazines,^22^, ^23^, ^24^ pyrimidines,^25^ amino acids,^26^ diazirines,^27^ and acetonitrile.^28^ It hyperpolarizes not only their ^1^H nuclei but the wide array of heteronuclei that they contain.^19^, ^24^, ^25^, ^29^, ^30^

It has recently been shown that the selective, partial ^2^H labelling of substrates such as pyridine,^13^, ^31^ nicotinamide and methyl nicotinate greatly extends the *T*_1_ relaxation times of the remaining ^1^H nuclei through inhibition of scalar relaxation processes.^14^, ^32^, ^33^ In addition, through ^2^H labelling of the IMes ligand of the iridium catalyst, the relaxation times of the bound substrate increase which further results in a dramatic improvement in the ^1^H hyperpolarization levels that can be achieved through SABRE. In fact, they exceed 50% polarization when 5 bar of *para*hydrogen is used in conjunction with a co‐ligand.^14^ In this current work, we seek to report further developments that employ this ^2^H labelling strategy by applying it to the related compounds pyridine, methyl isonicotinate, and isonicotinamide. We seek to ascertain its effect on the efficacy of the resulting ^1^H and ^13^C hyperpolarization levels that can be achieved through SABRE. For synthetic procedures and characterisation data please see Supporting information.

## 2 RESULTS AND DISCUSSION

**Figure 1:**
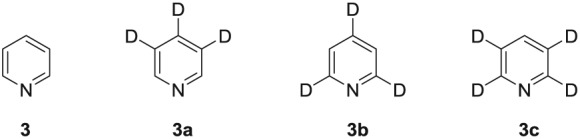
^2^H‐labelled pyridine isotopologues used in this study

Pyridine (**3**) was one of the first compounds to be hyperpolarized by the SABRE method.^7^, ^12^, ^15^ Because of its relatively simple structure, we recognized that there was an opportunity to investigate the effect of selective ^2^H labelling on the level of hyperpolarization. A selection of partially deuterated pyridines shown in Figure 1 were therefore synthesized.^34^, ^35^ This approach gave us access to three isotopologues of pyridine, **3a**, **3b**, and **3c**, in which ^1^H nuclei are retained at the *ortho*, *meta*, and *para* positions of the ring, respectively.

**Figure 2:**
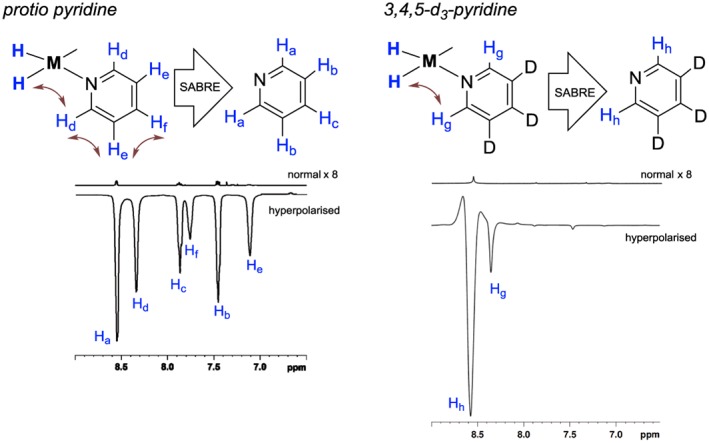
Left: two single scan ^1^H NMR spectra that illustrate pyridine (**3**) signals (attributed according to inset scheme) that result after SABRE (hyperpolarised) and when the polarization level matches that for thermodynamic equilibrium (normal). Right: two similar single scan ^1^H NMR spectra employing **3a**. The normal traces are shown with a ×8 vertical expansion relative to the hyperpolarized trace

When *protio*‐pyridine **3** (20 mM, 4 eq.) is mixed with [IrCl(COD)IMes] (5 mM) and *para*hydrogen in ethanol‐*d*_6_ and shaken at 65 gauss for 10 s followed by immediate transport into the spectrometer for detection, the signal for H_a_ in Figure 2 appears with an intensity that is approximately 2,400 times larger than that observed under Boltzmann conditions in the corresponding thermally equilibrated control measurement. In contrast, the corresponding signals for H_b_ and H_c_ are 1,120 and 1,258 times larger than those of the corresponding reference signals (at 9.4 T). Figure 2 (left) illustrates a typical measurement. The new signal intensity reflects the detection of 3.9% H_a_ polarization, created after just 10 s of exposure to 3 bar of *para*hydrogen. Furthermore, we note that if 20 s were needed to repeat the control measurement which uses thermally equilibrated polarization levels, it would take 1,333 days of data averaging to reproduce the hyperpolarized signal intensity. It is for this reason that hyperpolarization reflects a method that could transform clinical diagnosis as it may facilitate the facile detection of markers that probe underlying physiology.^36^

When the agent 3,4,5‐*d*_3_‐pyridine (**3a**) was employed under identical conditions to those described for **3**, the signal enhancement seen for the remaining *ortho* protons increases to 4,166 (6.7% polarization), shown in Table 1. However, upon moving to 2,4,6‐*d*_3_‐pyridine (**3b**), the remaining *meta* protons exhibit a smaller gain associated with an enhancement of just 541 (0.9% polarization). Finally, 2,3,5,6‐*d*_4_‐pyridine (**3c**) results in very little signal gain with its remaining proton showing a 163‐fold enhancement (0.51% polarization). These values are the average of three distinct measurements and are hence reproducible.

**Table 1:** *T*_1_ relaxation times and polarization levels achieved for pyridines **3** through SABRE in the specified solvent. Conditions: 20 mM substrate, 5 mM [IrCl(COD)IMes], activated with 3 bar *p‐*H_2_. *o* = *ortho*, *m* = *meta*, and *p* = *para*

Substrate	Methanol‐*d*_4_			Ethanol‐*d*_6_		
	Site *T*_1(no cat.)_/s	Site *T*_1(with cat.)_/s	Polarization level (%)	Site *T*_1(no cat.)_ / s	Site *T*_1(with cat.)_ /s	Polarization level (%)
(1) 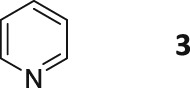	*o*—28.1 *m*—26.2 *p*—33.8	*o*—1.5 *m*—5.2 *p*—5.5	*o*—4.3 ± 0.3 *m*—3.4 ± 0.1 *p*—5.4 ± 0.1	*o*—20.7 *m*—18.2 *p*—26.7	*o*—3.2 *m*—4.7 *p*—5.3	*o*—3.9 ± 0.2 *m*—1.8 ± 0.1 *p*—4.0 ± 0.1
(2) 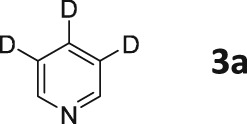	*o*—51.2	*o*—5.2	*o*—5.8 ± 0.4	*o*—72.4	*o*—4.2	*o*—6.7 ± 0.4
(3) 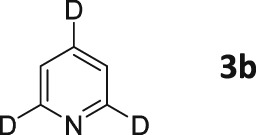	*m*—113.2	*m*—46.7	*m*—0.6 ± 0.1	*m*—72.6	*m*—13.8	*m*—0.9 ± 0.1
(4) 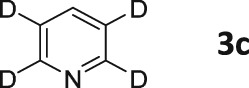	*p*—84.7	*p*—67.0	*p*–0.54 ± 0.12	*p*—57.5	*p*—25.0	*p*—0.51 ± 0.07

The lower polarization levels seen for **3b** and **3c** can be rationalized as the result of the fact that their protons are magnetically isolated from those of the hydride ligands in the iridium catalyst. Hence, smaller *J*‐couplings are involved in the polarization transfer step and less effective SABRE transfer results.^37^ By comparison, **3a** features two *ortho* ^1^H nuclei that couple more strongly to the hydride ligands in the SABRE‐catalyst and they are therefore able to more readily receive magnetization. An analogous trend was found when methanol‐*d*_4_ was used as the solvent, and in this case, a polarization level of 5.8% could be achieved with **3a**.

The reduction in hydride‐substrate *J*‐coupling is somewhat offset by relaxation changes. At 400 MHz, in degassed ethanol‐*d*_6_, *protio*‐pyridine (**3**) was found to exhibit *T*_1_ relaxation times of between 18.2 and 26.7 s (Table 1). Its isotopologue **3a**, however, has relaxation times for the two *ortho* protons of 72.4 s, whereas in **3b**, the *meta* protons exhibit a 72.6 s value and in **3c**, the *para* proton has a T_1_ of 57.5 s. The values determined in the ^2^H‐labelled isotopologues therefore reflect a dramatic improvement on those of the unlabelled **3**. We note, however, that under the catalytic conditions used in SABRE, interactions with the iridium catalyst act to reduce the observed *T*_1_ values. This is because the free and bound forms are in equilibrium and the observed value therefore reflects a weighted average of the two. For unlabelled pyridine, the new values lie between 3.2 and 5.3 s, whereas for **3a**, **3b**, and **3c**, they are 4.2, 13.8, and 25.0 s, respectively. As percentages, the reductions caused by the presence of the catalyst in solution are therefore 94%, 81%, and 56%, respectively. This serves to demonstrate that the effect is most strongly manifest in the *ortho* protons, which exhibit the strongest spin‐spin coupling to the hydride ligands when bound to the iridium. Although **3b** exhibits a better *T*_1_ value for the *meta* position, it fails to receive strong magnetization through SABRE, a feature that is further demonstrated for the *para* proton of **3c**. Again, analogous relaxation and polarization trends were observed in methanol‐*d*_4_ solution. However, relaxation times for **3b** and **3c** were significantly extended in methanol‐*d*_4_, compared to ethanol‐*d*_6_.

Hence, for optimal SABRE activity we can conclude that it is desirable to locate a proton next to the catalyst binding site, where *J*_HH_ is maximized. However, we tension this need with the fact that such an arrangement will also reduce the effective lifetime of the polarization under catalytic conditions. Clearly, the interplay between the *T*_1_ of the proton site and the efficacy of polarization transfer must therefore be carefully considered when designing an optimized agent.

**Figure 3:**
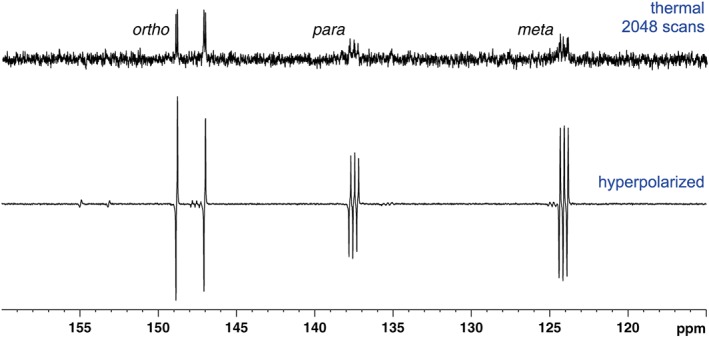
*Top*: Thermal ^13^C NMR spectrum of **3a** (100 mM) and SABRE catalyst (5 mM) in methanol‐*d*_4_ after 2,048 scans. Bottom: single‐scan hyperpolarized ^13^C NMR spectrum

A series of hyperpolarized ^13^C measurements were then conducted using 3,4,5‐*d*_3_‐pyridine (**3a**) in the presence of **1** for a 20::1 loading after SABRE at approximately 0.5 G. The resulting fully coupled single‐scan ^13^C hyperpolarized NMR spectrum is shown in Figure 3 alongside its thermally equilibrated ^13^C counterpart, which is a 2,048 scan average. A significant increase in the ^13^C resonances' signal‐to‐noise ratios is clearly observed in the hyperpolarized spectrum. Furthermore, the *ortho* peak (148 ppm) is observed as an antiphase doublet of doublets, whereas the *meta* (124 ppm) and *para* (137 ppm) peaks are split into antiphase triplets of doublets. This is consistent with the creation of *I*_z_ *S*_z_ terms, where the antiphase component is associated with the small, indirect *J*_HC_ coupling. The *meta* and *para* signals are associated with the detection of a ^13^CD signal for the *meta* and *para* sites, which accounts for the in‐phase 1:1:1 splitting.

**Figure 4:**
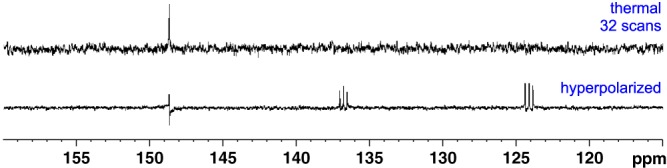
Top: thermal ^13^C{^1^H} NMR spectrum of **3a** (100 mM) and SABRE catalyst (5 mM) in methanol‐*d*_4_ over 32 scans. Bottom: single‐scan hyperpolarized ^13^C{^1^H} NMR spectrum

When a similar experiment was recorded with concurrent ^1^H decoupling a much weaker signal was observed (Figure 4). This confirms that the dominant terms that are created under SABRE at 60 G are indeed *I*_z_ *S*_z_ based rather than *S*_z_. We expect that the levels of signal gain that are observed may be improved through the incorporation of a ^15^N label, which has recently been shown to reduce ^13^C polarization transfer losses due to quadrupolar relaxation.^38^

**Scheme 2:**
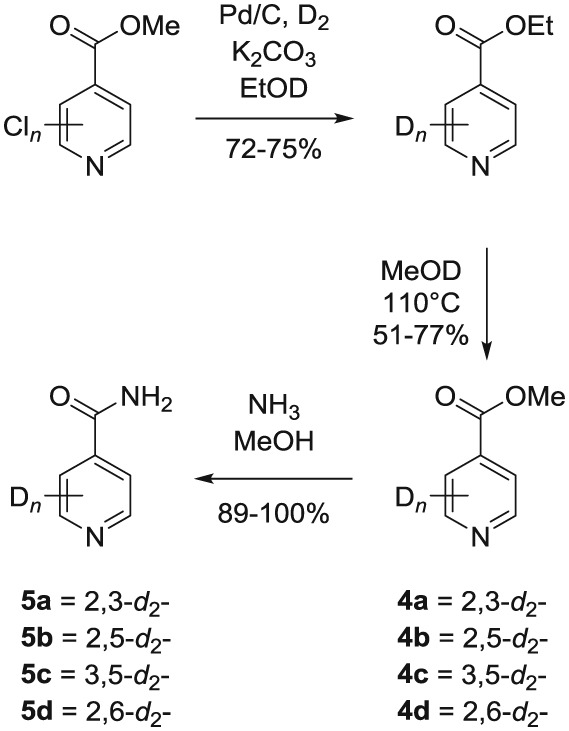
Synthetic route to the doubly ^2^H‐labelled methyl isonicotinates **4** and isonicotinamides **5**

Following these observations, we turned our attention to the *para‐*substituted pyridine derivatives, methyl isonicotinate (**4**) and isonicotinamide (**5**). A range of doubly deuterated isotopologues of these compounds were synthesized according to Scheme 2, and the results of the related NMR studies are shown in Table 2. These substrates provide a range of ^1^H spin systems, and we expected them to exhibit markedly different hyperpolarization characteristics. This reflects the fact that **4a** and **5a** possess pairs of *ortho* and *meta* ^1^H nuclei that exhibit a mutual three‐bond coupling, in contrast, **4b** and **5b** contain two weakly coupled *ortho* and *meta* nuclei (5‐bond coupling), whereas **4c**, **5c**, **4d**, and **5d** contain pairs of equivalent *ortho* and *meta* nuclei, respectively.

**Table 2:** *T*_1_ relaxation times (s) and polarization levels found for the methyl isonicotinates **4** and the isonicotinamides **5**. Conditions: 20 mM substrate, 5 mM [IrCl(COD)IMes], activated with 3 bar *p‐*H_2_. *o* = *ortho* and *m* = *meta*

Substrate	Methanol‐*d*_4_		
	Site *T*_1(no cat.)_/s	Site *T*_1(with cat.)_/s	Polarization level (%)
(5) 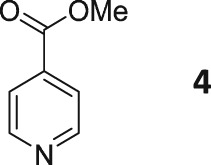	*o*—14.2 *m*—14.1	*o*—1.9 *m*—3.9	*o*—4.8 ± 0.1 *m*—1.0 ± 0.1
(6) 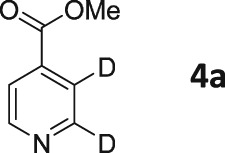	*o*—9.6 *m*—9.6	*o*—2.9 *m*—5.8	*o*—2.2 ± 0.1 *m*—1.4 ± 0.1
(7) 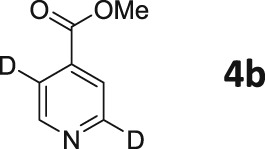	*o*—61.9 *m*—61.2	*o*—3.5 *m*—21.4	*o*—5.4 ± 0.4 *m*—5.6 ± 0.3
(8) 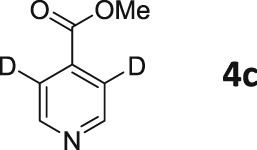	*o*—70.7	*o*—4.1	*o*—7.4 ± 0.3
(9) 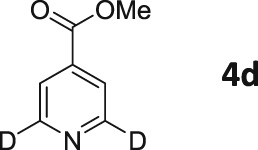	*m*—72.8	*m*—16.1	*m*—7.4 ± 0.4
(10) 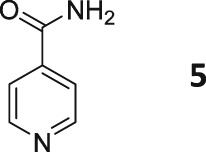	*o*—8.2 *m*—8.3	*o*—1.7 *m*—3.5	*o*—2.6 ± 0.2 *m*—0.2 ± 0.02
(11) 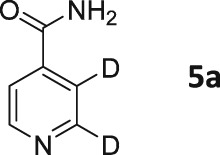	*o*—7.4 *m*—8.3	*o*—2.3 *m*—4.1	*o*—3.5 ± 0.3 *m*—0.4 ± 0.1
(12) 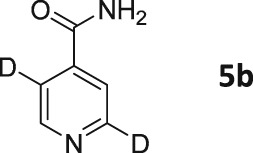	*o*—18.2 *m*—42.7	*o*—4.2 *m*—16.0	*o*—11.0 ± 0.5 *m*—11.1 ± 0.5
(13) 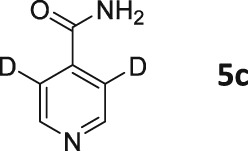	*o*—23.4	*o*—2.9	o—5.8 ± 0.7
(14) 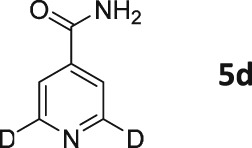	*m*—47.2	*m*—8.3	*m*—4.2 ± 0.1

**Figure 5:**
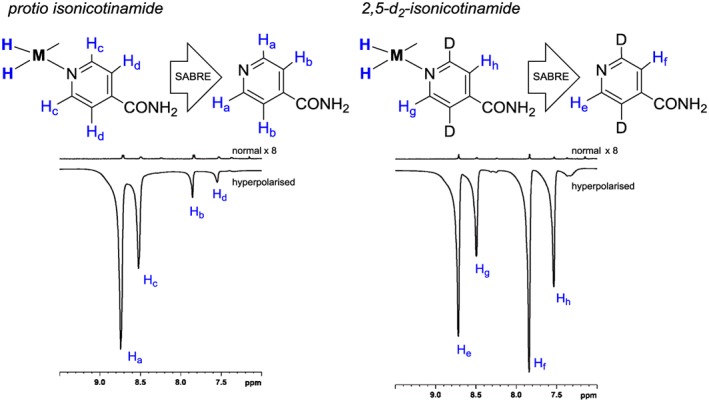
Left: single scan ^1^H NMR spectra for hyperpolarized and fully relaxed isonicotinamide **5** at 400 MHz. Right: corresponding single scan ^1^H NMR spectra of **5b**. The normal traces are shown with a ×8 vertical expansion relative to the hyperpolarized traces

For these ester and amide substrates, deuteration at the 2‐ and 3‐positions (**4a** and **5a**) resulted in limited changes in their relaxation times relative to their ^1^H counterpart and somewhat similar polarization levels as a result of SABRE were observed (Table 2). These results indicate that relaxation within these molecules is driven by interactions between the adjacent ^1^H nuclei. Analogues **b**–**d**, with more isolated ^1^H spin systems, might therefore be predicted to have higher *T*_1_ values. In support of this, we found that deuteration at the 2‐ and 5‐positions (**4b** and **5b**) does indeed result in significantly improved relaxation times and SABRE polarization levels (Figure 5). Now, the *T*_1_ values are over 60 s for methyl 2,5‐*d*_2_‐isonicotinate **4b**, which compare to 14 s for protio **4**. In the case of isonicotinamide **5b**, the SABRE polarization levels improve from the original 2.6% and 0.2% levels for the *ortho* and *meta* positions of **5** (in methanol‐*d*_4_), respectively, to 11.0% and 11.1%, respectively. In this case, the extension of the *T*_1_ values determined for the *meta* proton in the presence of the SABRE catalyst greatly assists in increasing the observed polarization level of the proton at this position.

In general, compounds with only *ortho* ^1^H nuclei (**4c** and **5c**) gave slightly improved levels of polarization and *T*_1_ values that compare to those of the parent, but still suffer from short relaxation times in the presence of the SABRE catalyst. This is consistent with the results outlined earlier for **3a**. Compounds **4d** and **5d**, with only *meta* ^1^H nuclei now give higher *T*_1_ values, but as expected, the improvement in achieved polarization level is minimal, in accordance with the weaker *J*‐coupling that connects them to hydride ligands in the catalyst.

These trends confirm our earlier conclusions that substrates containing a ^1^H nucleus at the *ortho* position result in efficient polarization transfer and that ^1^H nuclei that are isolated from each other, and the hydride ligands of the metal catalyst, lead to increased relaxation times under SABRE. These changes contribute cooperatively to produce a strong, long‐lived hyperpolarized signal.

**Figure 6:**
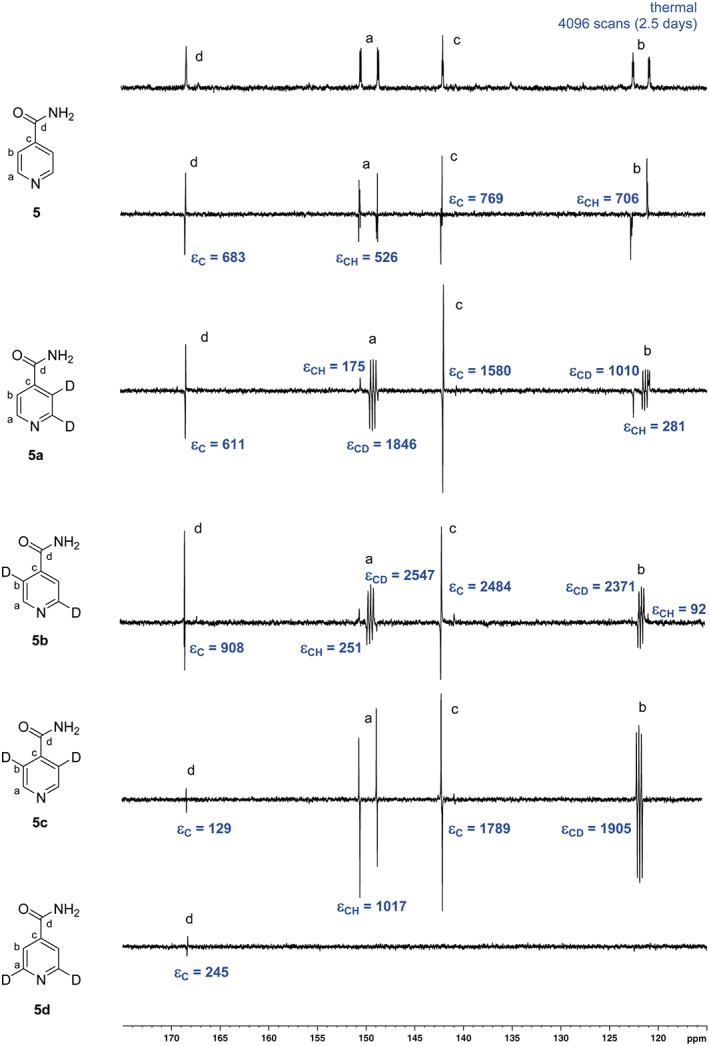
Series of hyperpolarized single‐scan ^13^C NMR spectra of **5**–**5d** (100 mM) and [IrCl(COD)(IMes)] (5 mM) in methanol‐*d*_4_ under *p*‐H_2_ after transfer at 0.5 G. Thermally polarized reference ^13^C NMR spectrum (top), acquired over 4,096 scans. ε = enhancement factor, fold gain

Related hyperpolarized ^13^C NMR experiments on the labelled isonicotinamides (**5a**–**5d**) showed that superior signal enhancements could be achieved with these substrates (Figure 6). With a 20‐fold concentration of substrate to catalyst and polarization transfer at approximately 0.5 gauss, compound **5a** showed strong SABRE responses for the quaternary carbons, including those adjacent to the ^2^H labels. The remaining CH positions showed a much lower, but detectable signal enhancement, an effect which is predicted to be due to an increased rate of relaxation. Compound **5b** produced a similar outcome; the higher levels of ^1^H polarization enabling increased magnetization transfer to be relayed into the most distant quaternary carbon. In the case of compound **5c**, a clear SABRE signal was observed for all carbons on the aromatic ring, notably including that of the CH at the *ortho* position. The carbonyl carbon showed significantly smaller polarization, as only a weak ^4^ *J*_CH_ coupling to the ortho proton is possible from that site for transfer. Analogue **5d** gave very little ^13^C polarization, with only the carbonyl position being visible. It should be noted that in all cases, the dominant peaks appear in antiphase due to the observation of *I*_z_ *S*_z_ derived states.

## 3 CONCLUSIONS

An investigation has been made into the effect of selectively incorporating ^2^H labels into a number of pyridine‐based substrates and the subsequent effect this change had on the observed hyperpolarization levels achieved through the SABRE process. We completed studies on three isotopologues of pyridine (**3**), four isotopologues of methyl isonicotinate (**4**), and four isotopologues of isonicotinamide (**5**). We find that harnessing hyperpolarization sites adjacent to the nitrogen centre of pyridine, which binds to the SABRE catalyst metal centre, is crucial for efficient polarization transfer through its stronger ^4^ *J*_HH_ coupling to the *para*hydrogen‐derived metal hydride ligands. Furthermore, we confirm that the presence of spin‐isolated hyperpolarization sites in these agents both increases signal lifetime through reduced relaxation and allows the detection of strongly hyperpolarized ^1^H responses. These changes also facilitate the detection of strong ^13^C NMR signals, most notably for the corresponding quaternary and CD positions. These signals typically appear with anti‐phase character due their origin in a heteronuclear longitudinal two spin order term (^1^H─^13^C) involving an indirect coupling. Agent **5b** produced the strongest carbonyl signal as a consequence of a ^3^ *J*_HC_ coupling, which must lead to limited internal peak cancelation due to its small value. The ^13^CH signals of **5c** are also dramatically stronger than those of **5a**, **5b**, and **5d** in accordance with a predicted long relaxation time. The spin isolation of the C─^13^CONH_2_ groups in **5c** and **5d** though acts to reduce its detectability, although the signal for C─^13^CONH_2_ is strongly visible in **5a** and **5b**. Hence, we conclude that a ^2^H‐labelling strategy can be used to control not only ^1^H signal gains but also those of ^13^C. We expect that this strategy will enable improvement in polarization in molecules containing other heteronuclei, such as ^15^N, and work is ongoing to achieve this goal. Additionally, a detailed investigation into the mechanism of polarization transfer in molecules containing ^2^H nuclei would be of interest.

## Supporting information

Data S1 Supporting Information
