# Supplementary material for: Using 2H labelling to improve the NMR detectability of pyridine and its derivatives by SABRE
Source: Magn Reson Chem. 2018 Jan 25;56(7):663–71. doi: 10.1002/mrc.4703 (PMC6001449; doi:10.1002/mrc.4703)
Supplement: Supplementary file 1 — Data S1 Supporting Information [file MRC-56-663-s001.docx]

**Using ^2^H Labelling to Improve the NMR detectability of a Series of Pyridines by SABRE**

Philip Norcott, Michael J. Burns, Peter J. Rayner, Ryan E. Mewis and Simon B. Duckett*

Centre for Hyperpolarization in Magnetic Resonance, Department of Chemistry, University of York, York YO10 5NY, U.K.

**Contents**

**1 Experimental procedures and Compound Characterisation Data**

**1.1 General**

**1.2 Synthetic Methods and Characterization Data**

**2 NMR polarization transfer experiment data**

**2.1** **Polarization transfer methods**

**2.2 ^1^H Polarization factors**

**1 Experimental Procedures and Compound Characterisation Data**

**1.1 General**

Water is distilled water. Brine refers to a saturated aqueous solution of NaCl. THF was freshly distilled from sodium and benzophenone ketyl or dried using a Grubbs solvent purification system. Petrol refers to the fraction of petroleum ether boiling in the range 40-60 °C. All reactions were carried out under O_2_-free Ar or N_2_ using oven-dried and/or flame-dried glassware.

Flash column chromatography was carried out using Fluka Chemie GmbH silica (220-440 mesh). Reverse-phase flash column chromatography was carried out using a Biotage Isolera with a SNAP-C_18_-12g cartridge eluting with H_2_O-MeCN containing 0.1% NH_4_OH. Thin layer chromatography was carried out using Merck F_254_ aluminium-backed silica plates. ^1^H (400 MHz) and ^13^C (100.6 MHz) NMR spectra were recorded on a Bruker-400 instrument with an internal deuterium lock. Chemical shifts are quoted as parts per million and referenced to CHCl_3_ (δ_H_ 7.27), (CH_3_)_2_SO (δ_H_ 2.54), CDCl_3_ (δ_C_ 77.0) or (CD_3_)_2_SO (δ_C_ 40.45). ^13^C NMR spectra were recorded with broadband proton decoupling. ^13^C NMR spectra were assigned using DEPT experiments. Coupling constants (*J*) are quoted in Hertz. Electrospray high and low resolution mass spectra were recorded on a Bruker Daltronics microOTOF spectrometer.

**1.2 Synthetic Methods and Compound Characterization Data**

***d*_5_-Pyridine *N*-oxide S1**

A solution of pyridine *N*-oxide (2.0 g, 21 mmol) and potassium carbonate (2.0 g, 14 mmol) in D_2_O (20 mL) was heated to 190 °C in an autoclave for 5 hours. The reaction mixture was allowed to cool and concentrated under reduced pressure. D_2_O (15 mL) was added and the solution heated to 190 °C for a further 5 hours. The reaction mixture was allowed to cool and extracted with CH_2_Cl_2_ (10 × 20 mL), dried over Na_2_SO_4_ and concentrated under reduced pressure to give *d*_5_-pyridine *N*-oxide **S1** (2.36 g, 75%) as a white powder; **^13^C NMR** (101 MHz, CDCl_3_) δ 138.5 (t, *J* = 27.8 Hz), 132.1 (t, *J* = 26.5 Hz), 126.8 (t, *J* = 26.1 Hz).

**3,4,5-*d*_3_-Pyridine *N*-oxide S2**

*d*_5_-Pyridine *N*-oxide (2.36 g, 23.6 mmol) was dissolved in 10% aqueous Na_2_CO_3_ solution (150 mL) and heated to reflux for 12 hours. The solution was allowed to cool and extracted with CH_2_Cl_2_ (5 × 20 mL), dried over Na_2_SO_4_ and concentrated under reduced pressure to give 3,4,5-*d*_3_-pyridine *N*-oxide **S2** (1.16 g, 50%) as a white solid; **^1^H NMR** (400 MHz, CDCl_3_) δ 8.64 (s, 2H); **^13^C NMR** (101 MHz, CDCl_3_) δ 140.1 (s), 132.4 (t, *J* = 26.1 Hz), 127.6 (t, *J* = 26.0 Hz).

**3,4,5-*d*_3_-Pyridine 3a**

To a solution of 3,4,5-*d*_3_-pyridine *N*-oxide (1.16 g, 11.8 mmol) in CH_2_Cl_2_ (10 mL) at 0 °C was added phosphorus trichloride (2.0 mL, 23 mmol, 2 eq.) dropwise. The reaction mixture was heated to reflux for 1 hour. The solution was allowed to cool to room temperature, poured over crushed ice and basified with NaOH solution (10 M). The organic layer was collected and the aqueous layer extracted with CH_2_Cl_2_ (5 × 10 mL). The combined organic fractions were dried over Na_2_SO_4_ and concentrated under reduced pressure. The oily residue was transferred to a vacuum distillation apparatus and the distillate formed at 115 °C collected to give 3,4,5-*d*_3_-pyridine **3a** (0.52 g, 54%) as a colourless liquid; **^1^H NMR** (400 MHz, CDCl_3_) δ 8.56 (s, 2H); **^13^C NMR** (101 MHz, CDCl_3_) δ 149.74 (s), 135.3 (t, *J* = 24.6 Hz), 123.2 (t, *J* = 25.2 Hz).

**2,4,6-*d*_3_-Pyridine 3b**

To a suspension of 2,4,6-trichloropyridine (1.0 g, 5.5 mmol) and K_2_CO_3_ (2.3 g, 17 mmol, 3 eq.) in THF (5 mL) and D_2_O (5 mL) in a 30 mL Parr reactor was added 5% Pd/C (100 mg, 10 wt%). The reactor was sealed, purged with N_2_, then pressurised with D_2_ (8 bar). The reaction mixture was stirred at room temperature for 18 hours. The pressure was released and the suspension was filtered through Celite and washed with diethyl ether (20 mL). The filtrate was dried over MgSO_4_ then acidified with HCl (2 M in diethyl ether, 4 mL). The resulting suspension was decanted to leave an oily residue which solidified under a stream of nitrogen. This solid was dissolved in methanol-*d*_4_ (4 mL) and passed through a plug of basic alumina to give a solution of 2,4,6-*d*_3_-pyridine **3b** (0.21 M in methanol-*d*_4_); **^1^H NMR** (400 MHz, CDCl_3_) *δ* (ppm) 7.44 (s, 2H); **^13^C NMR** (101 MHz, CDCl_3_) *δ* (ppm) 149.5 (t, *J* = 27.5 Hz), 138.2 (t, *J* = 25.0 Hz), 125.3 (s); **MS** (ESI) *m/z* 83 [(M + H)^+^, 100]. The concentration was determined by quantitative NMR with potassium phthalate monobasic as an internal standard.

**2,3,5,6-*d*_4_-Pyridine 3c**

To a suspension of 2,3,5,6-tetrachloropyridine (1.0 g, 4.6 mmol) and K_2_CO_3_ (2.6 g, 19 mmol, 4 eq.) in THF (5 mL) and D_2_O (5 mL) in a 30 mL Parr reactor was added 5% Pd/C (100 mg, 10 wt%). The reactor was sealed, purged with N_2_, then pressurised with D_2_ (8 bar). The reaction mixture was stirred at room temperature for 4 hours. The pressure was released and the suspension was filtered through Celite and washed with diethyl ether (20 mL). The filtrate was dried over MgSO_4_ then acidified with HCl (2 M in diethyl ether, 4 mL). The resulting suspension was decanted to leave an oily residue which solidified under a stream of nitrogen. This solid was dissolved in methanol-*d*_4_ (4 mL) and passed through a plug of basic alumina to give a solution of 2,3,5,6-*d*_4_-pyridine **3c** (0.16 M in methanol-*d*_4_); **^1^H NMR** (400 MHz, CDCl_3_) *δ* (ppm) 7.85 (s, 1H); **^13^C NMR** (101 MHz, CDCl_3_) *δ* (ppm) 149.4 (t, *J* = 27.2 Hz), 138.2 (s), 125.1 (t, *J* = 25.3 Hz); **MS** (ESI) *m/z* 84 [(M + H)^+^, 100]. The concentration was determined by quantitative NMR with potassium phthalate monobasic as an internal standard.

**Methyl 2,5-dichloroisonicotinate S3**

To a solution of 2,5-dichloroisonicotinic acid (500 mg, 2.6 mmol) in methanol (5 mL) and diethyl ether (5 mL) was added (trimethylsilyl)diazomethane solution (2.6 mL, 2.0 M in diethyl ether, 2 eq.). The reaction mixture was stirred at room temperature for 30 minutes, then concentrated under reduced pressure and purified by flash column chromatography with 4:1 petrol-EtOAc as eluent to give methyl 2,5-dichloroisonicotinate **S3** (518 mg, 97%) as a colourless solid; **^1^H NMR** (400 MHz, CDCl_3_) *δ* 8.43 (s, 1H), 7.65 (s, 1H), 3.93 (s, 3H); **^13^C NMR** (101 MHz, CDCl_3_) *δ* 163.2 (s), 150.9 (s), 149.9 (s), 139.2 (s), 129.1 (s), 125.1 (s), 53.3 (s); **MS** (ESI) *m/z* 206 [(M + H)^+^, 100], 208 [84]; **HRMS** (ESI) *m/z* [M + H]^+^ calculated for C_7_H_6_Cl_2_NO_2_ 205.9770, found 205.9766 (+2.9 ppm error).

**Ethyl 2,5-*d*_2_-isonicotinate S4**

To a suspension of methyl 2,5-dichloroisonicotinate (500 mg, 2.4 mmol) and K_2_CO_3_ (850 mg, 6.2 mmol, 2.5 eq.) in EtOD (10 mL) in a 30 mL Parr reactor was added 5% Pd/C (50 mg, 10 wt%). The reactor was sealed, purged with N_2_, then pressurised with D_2_ (8 bar). The reaction mixture was stirred at room temperature for 4 hours. The pressure was released and the suspension was filtered through Celite and washed with EtOH. The filtrate was concentrated under reduced pressure and purified by flash column chromatography with 1:1 petrol-EtOAc as eluent to give ethyl 2,5-*d*_2_-isonicotinate **S4** (268 mg, 72%) as a colourless oil; **^1^H NMR** (400 MHz, CDCl_3_) *δ* 8.75 (s, 1H), 7.82 (s, 1H), 4.39 (q, *J* = 7.1 Hz, 2H), 1.39 (t, *J* = 7.1 Hz, 3H); **^13^C NMR** (101 MHz, CDCl_3_) *δ* 165.2 (s), 150.6 (s), 150.3 (t, *J* = 27.3 Hz), 137.6 (s), 122.8 (s), 122.7 (t, *J* = 25.6 Hz), 61.9 (s), 14.3 (s); **MS** (ESI) *m/z* 154 [(M + H)^+^, 100], 126 [64]; **HRMS** (ESI) *m/z* [M + H]^+^ calculated for C_8_H_8_D_2_NO_2_ 154.0832, found 154.0832 (‒0.3 ppm error).

**Methyl 2,3-*d*_2_-isonicotinate 4a**

A suspension of ethyl 2,3-*d*_2_-isonicotinate (200 mg, 1.3 mmol) and K_2_CO_3_ (18 mg, 0.13 mmol) in MeOD (3 mL) was heated to 110 °C under microwave irradiation for 1 hour. The reaction mixture was concentrated under reduced pressure and purified by flash column chromatography with 2:1 petrol-EtOAc as eluent to give methyl 2,3-*d*_2_-isonicotinate **11a** (140 mg, 77%) as a colourless oil; **^1^H NMR** (400 MHz, CDCl_3_) *δ* 8.70 (d, *J* = 5.2 Hz, 1H), 7.84 (d, *J* = 5.2 Hz, 1H), 3.92 (s, 3H); **^13^C NMR** (101 MHz, CDCl_3_) *δ* 166.4 (s), 151.2 (s), 150.8 (t, *J* = 27.8 Hz), 139.1 (s), 124.2 (s), 123.8 (t, *J* = 26.0 Hz), 53.3 (s); **MS** (ESI) *m/z* 140 [(M + H)^+^, 100], 126 [16]; **HRMS** (ESI) *m/z* [M + H]^+^ calculated for C_7_H_6_D_2_NO_2_ 140.0675, found 140.0675 (‒1.4 ppm error).

**Methyl 2,5-*d*_2_-isonicotinate 4b**

A suspension of ethyl 2,5-*d*_2_-isonicotinate (268 mg, 1.8 mmol) and K_2_CO_3_ (25 mg, 0.18 mmol) in MeOD (3 mL) was heated to 110 °C under microwave irradiation for 1 hour. The reaction mixture was concentrated under reduced pressure and purified by flash column chromatography with 2:1 petrol-EtOAc as eluent to give methyl 2,5-*d*_2_-isonicotinate **11b** (124 mg, 51%) as a colourless oil; **^1^H NMR** (400 MHz, CDCl_3_) *δ* 8.74 (s, 1H), 7.80 (s, 1H), 3.92 (s, 3H); **^13^C NMR** (101 MHz, CDCl_3_) *δ* 165.6 (s), 150.6 (s), 150.3 (t, *J* = 27.8 Hz), 137.2 (s), 122.7 (s), 122.6 (t, *J* = 25.7 Hz), 52.8 (s); **MS** (ESI) *m/z* 140 [(M + H)^+^, 100]; **HRMS** (ESI) *m/z* [M + H]^+^ calculated for C_7_H_6_D_2_NO_2_ 140.0675, found 140.0680 (‒3.5 ppm error).

**Methyl 2,3-dichloroisonicotinate S5**

To a solution of 2,3-dichloroisonicotinic acid (500 mg, 2.6 mmol) in methanol (5 mL) and diethyl ether (5 mL) was added (trimethylsilyl)diazomethane solution (2.6 mL, 2.0 M in diethyl ether, 2 eq.). The reaction mixture was stirred at room temperature for 30 minutes, then concentrated under reduced pressure and purified by flash column chromatography with 4:1 petrol-EtOAc as eluent to give methyl 2,3-dichloroisonicotinate **S5** (430 mg, 80%) as a colourless solid; **^1^H NMR** (400 MHz, CDCl_3_) *δ* 8.21 (d, *J* = 4.9 Hz, 1H), 7.39 (d, *J* = 4.9 Hz, 1H), 3.82 (s, 3H); **^13^C NMR** (101 MHz, CDCl_3_) *δ* 163.6 (s), 150.8 (s), 146.8 (s), 140.1 (s), 128.1 (s), 122.6 (s), 52.9 (s); **MS** (ESI) *m/z* 206 [(M + H)^+^, 100], 208 [57]; **HRMS** (ESI) *m/z* [M + H]^+^ calculated for C_7_H_6_Cl_2_NO_2_ 205.9770, found 205.9775 (‒2.8 ppm error).

**Ethyl 2,3-*d*_2_-isonicotinate S6**

To a suspension of methyl 2,3-dichloroisonicotinate (400 mg, 1.9 mmol) and K_2_CO_3_ (670 mg, 4.8 mmol, 2.3 eq.) in EtOD (10 mL) in a 30 mL Parr reactor was added 5% Pd/C (40 mg, 10 wt%). The reactor was sealed, purged with N_2_, then pressurised with D_2_ (8 bar). The reaction mixture was stirred at room temperature for 2 hours. The pressure was released and the suspension was filtered through Celite and washed with EtOH. The filtrate was concentrated under reduced pressure and purified by flash column chromatography with 1:1 petrol-EtOAc as eluent to give ethyl 2,3-*d*_2_-isonicotinate **S6** (222 mg, 75%) as a colourless oil; **^1^H NMR** (400 MHz, CDCl_3_) *δ* 8.54 (d, *J* = 5.1 Hz, 1H), 7.61 (d, *J* = 5.1 Hz, 1H), 4.18 (q, *J* = 7.2 Hz, 2H), 1.17 (t, *J* = 7.2 Hz, 3H); **^13^C NMR** (101 MHz, CDCl_3_) *δ* 164.6 (s), 150.2 (s), 149.8 (t, *J* = 27.5 Hz), 137.2 (s), 122.5 (s), 122.1 (t, *J* = 25.8 Hz), 61.4 (s), 13.8 (s); **MS** (ESI) *m/z* 154 [(M + H)^+^, 100], 126 [48]; **HRMS** (ESI) *m/z* [M + H]^+^ calculated for C_8_H_8_D_2_NO_2_ 154.0832, found 154.0832 (+0.1 ppm error).

**Ethyl 3,5-*d*_2_-isonicotinate S7**

To a suspension of methyl 3,5-dichloroisonicotinate (500 mg, 2.4 mmol) and K_2_CO_3_ (850 mg, 6.2 mmol, 2.3 eq.) in EtOD (10 mL) in a 30 mL Parr reactor was added 5% Pd/C (50 mg, 10 wt%). The reactor was sealed, purged with N_2_, then pressurised with D_2_ (8 bar). The reaction mixture was stirred at room temperature for 2 hours. The pressure was released and the suspension was filtered through Celite and washed with EtOH. The filtrate was concentrated under reduced pressure and purified by flash column chromatography with 1:1 petrol-EtOAc as eluent to give ethyl 3,5-*d*_2_-isonicotinate **S7** (309 mg, 83%) as a colourless oil; **^1^H NMR** (400 MHz, CDCl_3_) *δ* 8.64 (s, 2H), 4.28 (q, *J* = 7.2 Hz, 2H), 1.27 (t, *J* = 7.2 Hz), 3H); **^13^C NMR** (101 MHz, CDCl_3_) *δ* 164.9 (s), 150.3 (s), 137.3 (s), 122.4 (t, *J* = 25.5 Hz), 61.6 (s), 14.0 (s); **MS** (ESI) *m/z* 154 [(M + H)^+^, 100], 126 [57]; **HRMS** (ESI) *m/z* [M + H]^+^ calculated for C_8_H_8_D_2_NO_2_ 154.0832, found 154.0834 (‒3.7 ppm error).

**Methyl 3,5-*d*_2_-isonicotinate 4c**

A suspension of ethyl 3,5-*d*_2_-isonicotinate (100 mg, 0.65 mmol) and K_2_CO_3_ (10 mg, 0.072 mmol) in MeOD (3 mL) was heated to 110 °C under microwave irradiation for 1 hour. The reaction mixture was concentrated under reduced pressure and purified by flash column chromatography with 1:1 petrol-EtOAc as eluent to give methyl 3,5-*d*_2_-isonicotinate **11c** (48 mg, 53%) as a colourless oil; **^1^H NMR** (400 MHz, CDCl_3_) *δ* 8.60 (s, 2H), 3.77 (s, 3H); **^13^C NMR** (101 MHz, CDCl_3_) *δ* 165.3 (s), 150.3 (s), 136.9 (s), 122.3 (t, *J* = 25.5 Hz), 52.4 (s); **MS** (ESI) *m/z* 140 [(M + H)^+^, 100]; **HRMS** (ESI) *m/z* [M + H]^+^ calculated for C_7_H_6_D_2_NO_2_ 140.0675, found 140.0675 (‒2.0 ppm error).

**Methyl 2,6-*d*_2_-isonicotinate 4d**

To a suspension of methyl 2,6-dichloroisonicotinate (500 mg, 2.4 mmol) and Na_2_CO_3_ (850 mg, 6.2 mmol, 2.5 eq.) in EtOD (10 mL) and D_2_O (1 drop) in a 30 mL Parr reactor was added 5% Pd/C (50 mg, 10 wt%). The reactor was sealed, purged with N_2_, then pressurised with D_2_ (8 bar). The reaction mixture was stirred at room temperature for 1 hour. The pressure was released and the suspension was filtered through Celite and washed with EtOH. The solvent was removed under reduced pressure and to the residue was added MeOD (3 mL) and K_2_CO_3_ (35 mg, 0.25 mmol). The reaction mixture was heated to 110 °C under microwave irradiation for 1 hour, then concentrated under reduced pressure and purified by flash column chromatography with 1:1 petrol-EtOAc as eluent to give methyl 2,6-*d*_2_-isonicotinate **11d** (113 mg, 39%) as a colourless oil; **^1^H NMR** (400 MHz, CDCl_3_) *δ* 7.74 (s, 2H), 3.86 (s, 2H); **^13^C NMR** (101 MHz, CDCl_3_) *δ* 165.5 (s), 150.2 (t, *J* = 27.7 Hz), 137.2 (s), 122.6 (s), 52.6 (s); **MS** (ESI) *m/z* 140 [(M + H)^+^, 100]; **HRMS** (ESI) *m/z* [M + H]^+^ calculated for C_7_H_6_D_2_NO_2_ 140.0675, found 140.0676 (‒0.9 ppm error).

**2,3-*d*_2_-isonicotinamide 5a**

A solution of methyl 2,3-*d*_2_-isonicotinate (40 mg, 0.29 mmol) in ammonia (5 mL, 7 M solution in methanol) was stirred at room temperature for 60 hours. The reaction mixture was concentrated and purified by flash chromatography with 1:9 MeOH-CH_2_Cl_2_ as eluent to give 2,3-*d*_2_-isonicotinamide **12a** (34 mg, 95%) as a colourless solid; **^1^H NMR** (400 MHz, CDCl_3_) *δ* 8.69 (d, *J* = 5.3 Hz, 1H), 7.83 (d, *J* = 5.3 Hz, 1H), 4.97 (s, 2H); **^13^C NMR** (101 MHz, CDCl_3_) *δ* 169.7 (s), 150.9 (s), 150.5 (t, *J* = 27.5 Hz), 143.3 (s), 123.1 (s), 122.7 (t, *J* = 25.6 Hz); **MS** (ESI) *m/z* 125 [(M + H)^+^, 100]; **HRMS** (ESI) *m/z* [M + H]^+^ calculated for C_6_H_5_D_2_N_2_O 125.0678, found 125.0676 (+1.8 ppm error)

**2,5-*d*_2_-isonicotinamide 5b**

A solution of methyl 2,5-*d*_2_-isonicotinate (30 mg, 0.22 mmol) in ammonia (5 mL, 7 M solution in methanol) was stirred at room temperature for 24 hours. The reaction mixture was concentrated and purified by flash chromatography with 1:9 MeOH-CH_2_Cl_2_ as eluent to give 2,5-*d*_2_-isonicotinamide **12b** (27 mg, 100%) as a colourless solid; **^1^H NMR** (400 MHz, CDCl_3_) *δ* 8.69 (s, 1H), 7.82 (s, 1H), 4.97 (s, 2H); **^13^C NMR** (101 MHz, CDCl_3_) *δ* 169.7 (s), 150.9 (s), 150.6 (t, *J* = 27.9 Hz), 143.3 (s), 123.1 (s), 122.9 (t, *J* = 26.8 Hz); **MS** (ESI) *m/z* 125 [(M + H)^+^, 100]; **HRMS** (ESI) *m/z* [M + H]^+^ calculated for C_6_H_5_D_2_N_2_O 125.0678, found 125.0680 (‒1.1 ppm error)

**3,5-*d*_2_-isonicotinamide 5c**

A solution of ethyl 3,5-*d*_2_-isonicotinate (30 mg, 0.20 mmol) in ammonia (5 mL, 7 M solution in methanol) was stirred at room temperature for 24 hours. The reaction mixture was concentrated and purified by flash chromatography with 1:9 MeOH-CH_2_Cl_2_ as eluent to give 3,5-*d*_2_-isonicotinamide **12c** (22 mg, 89%) as a colourless solid; **^1^H NMR** (400 MHz, CDCl_3_) *δ* 8.70 (s, 2H), 4.96 (s, 2H); **^13^C NMR** (101 MHz, CDCl_3_) *δ* 169.7 (s), 150.9 (s), 143.3 (s), 122.9 (t, *J* = 25.2 Hz); **MS** (ESI) *m/z* 125 [(M + H)^+^, 100]; **HRMS** (ESI) *m/z* [M + H]^+^ calculated for C_6_H_5_D_2_N_2_O 125.0678, found 125.0679 (+1.8 ppm error).

**2,6-*d*_2_-isonicotinamide 5d**

A solution of methyl 2,6-*d*_2_-isonicotinate (30 mg, 0.22 mmol) in ammonia (5 mL, 7 M solution in methanol) was stirred at room temperature for 24 hours. The reaction mixture was concentrated and purified by flash chromatography with 1:9 MeOH-CH_2_Cl_2_ as eluent to give 2,6-*d*_2_-isonicotinamide **12d** (25 mg, 93%) as a colourless solid; **^1^H NMR** (400 MHz, CDCl_3_) *δ* 7.82 (s, 2H), 4.90 (br s, 2H); **^13^C NMR** (101 MHz, CDCl_3_) *δ* 169.7 (s), 150.6 (t, *J* = 27.9 Hz), 143.5 (s), 123.0 (s); **MS** (ESI) *m/z* 125 [(M + H)^+^, 100]; **HRMS** (ESI) *m/z* [M + H]^+^ calculated for C_6_H_5_D_2_N_2_O 125.0678, found 125.0676 (+1.8 ppm error).

**2 NMR polarization transfer experiment data**

**2.1** **Polarization transfer methods**

The polarization transfer experiments that are reported were conducted in an NMR tube that was equipped with a Young’s Tap. Samples for these polarization transfer experiments were based on a 5 mM solution of [IrCl(COD)(NHC)] and 20 mM substrate in methanol-*d_4_*_,_ unless otherwise specified. The samples were degassed prior to the introduction of *para*hydrogen at a pressure of 3 bar. Samples were then shaken for 10 s in the specified fringe field of an NMR spectrometer before being rapidly transported into the magnet for subsequent interrogation by NMR spectroscopy.

**2.2 ^1^H Polarization factors**

For calculation of the enhancement of ^1^H NMR signals the following formula was used:

$$E=\frac{SI(pol)}{SI(unpol)}$$

Where, E = enhancement level, SI(pol) = signal of polarized sample, SI(unpol) = signal of unpolarized (reference) sample. Experimentally, both spectra were recorded on the same sample using identical acquisition parameters, including the receiver gain. The raw integrals of the relevant resonances in the polarized and unpolarized spectra were then used to determine the enhancement levels.
